# Procedure-Related Differences and Clinical Outcomes in Patients Treated with Percutaneous Coronary Intervention Assisted by Optical Coherence Tomography between New and Earlier Generation Software (Ultreon™ 1.0 Software vs. AptiVue™ Software)

**DOI:** 10.3390/jcdd9070218

**Published:** 2022-07-06

**Authors:** Rafał Januszek, Wojciech Siłka, Karol Sabatowski, Krzysztof Piotr Malinowski, Grzegorz Heba, Sławomir Surowiec, Michał Chyrchel, Łukasz Rzeszutko, Leszek Bryniarski, Andrzej Surdacki, Krzysztof Bartuś, Stanisław Bartuś

**Affiliations:** 1 Department of Cardiology and Cardiovascular Interventions, University Hospital, 30-688 Kraków, Poland; sabatowski.ka@gmail.com (K.S.); gheba@su.krakow.pl (G.H.); ssurowiec@wp.pl (S.S.); mchyrchel@gmail.com (M.C.); rzeszutko.lukasz@gmail.com (Ł.R.); l_bryniarski@poczta.fm (L.B.); andrzej.surdacki@uj.edu.pl (A.S.); stanislaw.bartus@uj.edu.pl (S.B.); 2 Students' Scientific Group, the Second Department of Cardiology, Jagiellonian University Medical College, 2 Jakubowskiego, 31-034 Kraków, Poland; wojciech.silka@student.uj.edu.pl (W.S.); krzysztof.piotr.malinowski@gmail.com (K.P.M.); 3 Institute of Cardiology, Jagiellonian University Medical College, 31-202 Kraków, Poland; krzysztofbartus@gmail.com; 4 Department of Cardiovascular Surgery and Transplantology, Jagiellonian University Medical College, John Paul II Hospital, 31-202 Kraków, Poland

**Keywords:** clinical outcomes, optical coherence tomography, percutaneous coronary intervention, procedural indices, software

## Abstract

(1) Introduction: Optical coherence tomography (OCT) intravascular imaging facilitates percutaneous coronary intervention (PCI). Software for OCT is being constantly improved, including the latest version Ultreon™ 1.0 Software (U) (Abbott Vascular, Santa Clara, CA, USA). In the current analysis, we aim to compare processing results, procedural indices as well as clinical outcomes in patients treated via PCI. This was conducted using earlier generation OCT imaging software versions (non-U) and the newest available one on the market (U). (2) Methods: The study comprised 95 subsequent and not selected patients (55 processed with U and 40 non-U). The non-U processings were transferred for evaluation by U software, while the comparison of OCT parameters, selected clinical and procedural indices was performed between groups. We further assessed clinical outcomes during the follow-up period, i.e., major adverse cardiovascular events (MACE) and predictors of stent expansion. (3) Results: We did not detect any differences in general features between either of the assessed groups at baseline. Non-U software was more often used for bare-metal stenting (*p* = 0.004), while PCIs in the U group demanded a greater number of stents (*p* = 0.03). The distal reference of external elastic lamina (EEL) diameter was greater in the non-U group (*p* = 0.02) with no concurrent differences in minimal (*p* = 0.27) and maximal (*p* = 0.31) stent diameter. It was also observed that MACE was more frequently observed in the non-U group (*p* = 0.01). Neither univariable (estimate: 0.407, 95%CI: (−3.182) − 3.998, *p* = 0.82) nor multivariable (estimate: 2.29, 95%CI: (−4.207) − 8.788, *p* = 0.5) analyses demonstrated a relationship between the type of software and stent expansion. (4) Conclusions: Improvement in the software for image acquisition and processing of OCT is not related to stent expansion. The EEL diameter is preferably used to select the distal stent diameter in newer software.

## 1. Introduction

The implementation of optical coherence tomography (OCT) in clinical practice has been reported by several studies. In them, improved outcomes have been demonstrated compared to angiography alone [1,2]. The advantage of OCT over intravascular ultrasound (IVUS) has been presented in the accuracy and reproducibility of measurement dimension [3].

The objective of the ILUMIEN I study was to define guidance parameters for stent optimization. Furthermore, the impact of OCT guidance on physician decision making, procedural findings and early clinical events was also assessed [4].

The ILUMIEN II study was a retrospective, post hoc comparison of OCT-guided vs. IVUS-guided stent expansion. Stent expansion was demonstrated to a similar degree in the case of the two modalities [5].

In the ILUMIEN III study, OCT-, IVUS- and angiography-guided PCI were compared [6]. It has been presented that decisions regarding stent diameter sizing are carried out on the basis of implementing the external elastic lamina (EEL) diameter measurement. This turned out to be appropriate. As a consequence, a similar stent expansion was obtained in comparison to IVUS-guided stenting [6,7]. Additionally, OCT-guided PCI has been known to cause greater post-PCI FFR in contrast to angio-guided PCI among non-ST segment elevation-acute coronary syndrome patients [8].

In the ILUMIEN III trial, a higher degree of OCT sensitivity was noted compared to IVUS and angiography. This regarded detection and prevention of major dissections and sections of stent malapposition.

Current European guidelines concerning non-ST segment elevation myocardial infarction state that intracoronary imaging usage (OCT and IVUS) may be the most precise option in uncertain situations when proving the occurrence of intramural hematoma or double lumen. Moreover, this may be key to formulate a proper diagnosis, especially among patients with suspicion of spontaneous coronary artery dissection, erosions, plaque ruptures or myocardial infarction with non-obstructive coronary arteries (MINOCA) [9].

The indication for using OCT in stent optimization has also been upgraded in the last European guidelines on myocardial revascularization from Class II b to Class IIa (Level B evidence) [10]. It has been suggested that IVUS and/or OCT should be taken into consideration when detecting stent-related mechanical problems causing restenosis (Class IIa (Level of evidence C)) [10]. European guidelines concerning ST-segment elevation myocardial infarction treatment mention OCT twice. They concern its usefulness in MINOCA diagnosis [11].

The most recent American guidelines on myocardial revascularization state that, among patients undergoing coronary stent implantation, OCT is a sensible substitute for IVUS in procedural guidance, apart from cases of ostial left main disease (recommendation class 2a as well as B-R evidence level) [12]. The implementation of algorithms in treatment could have potentially better outcomes while aiding the standardization of clinical practice [13].

The objective of the current research trial is to assess the correlation between the type of software used for the OCT imaging with regard to patients and procedural characteristics as well as procedural and clinical outcomes.

## 2. Methods

### 2.1. Patients

The current analysis included 95 successive and not selected patients presenting with stable angina. These patients were treated using PCI assisted by OCT. The treatment was carried out between October 2014 and March 2022, and was performed at the Department of Cardiology and Cardiovascular Interventions University Hospital in Kraków, Poland. There were 40 patients assisted by OCT equipped with earlier generation software: (1) ILUMIEN™ OPTIS-System™ (Abbott Vascular, Santa Clara, CA, USA) unit with AptiVue™ Software 5.2; and (2) ILUMIEN™ OPTIS-System™ (Abbott Vascular, Santa Clara, CA, USA) unit with 3D software.

On the other hand, the new generation (Ultreon™ 1.0 Software—group 55 patients) was assessed by the newest OPTIS™ Integrated Next Imaging System unit with Ultreon™ 1.0 Software (Abbott Vascular, Santa Clara, CA, USA). The only OCT catheter applied during the use of that equipment was the Dragonfly™ OPTIS™ Imaging Catheter (Abbott Vascular, Santa Clara, CA, USA).

### 2.2. Optical Coherence Tomography—Image Attainment and Processing

The procedure of acquiring intravascular images using the OCT probe was carried out in accordance with applicable international recommendations. The imaging was repeated at least twice, i.e., initially and during the final procedure stage, and in selected cases, i.e., chronic total occlusions or tight stenosis.

Examination was performed after the vessel was restored. This was often preceded by pre-dilatation to allow the blood to be rinsed out with contrast [14]. The Dragonfly™ OPTIS™ Imaging Catheter (Abbott Vascular, Santa Clara, CA, USA) was connected to the dedicated system (type depending on the time of acquisition: earlier generation vs. newer software). We used OCT-angiography co-registration in newer generations.

The pullback length was adjusted to the length of the visualized artery: 54 mm or 75 mm. In selected cases, it had to be repeated twice due to lesions longer than 75 mm. Pullback was performed automatically.

A contrast medium (Visipaque™, Iodixanol 320 mg/mL, GE Healthcare) was used to clear the lumen of blood, making it possible for the OCT to carry out imaging. Manual injections were adopted in all cases with 10 or 20 cc syringes, according to the type of artery (diameter and length of lesion). Each contrast flush was proceeded by 200 µg of nitroglycerin intracoronary injection. Flush clearance allowed for distinct distinction between the lumen and structure of the vessel.

OCT assessment was primarily performed according to the available software. Final assessment was carried out using the Ultreon™ 1.0 software after transferring from earlier generation equipment. The following algorithm was applied for analysis: Morphology, Length, Diameter, Medial Dissection, Apposition and Expansion (MLD-MAX). The proposed algorithm was recommended by Abbott Vascular. Several records were deleted due to lack of pre-stenting or post-stenting examination. None were deleted when, for example, probe damage occurred at baseline or during the examination. Furthermore, in the instance of using the OCT probe after PCI due to, e.g., complications, difficulties with procedure result evaluation or to visualize the endovascular procedure, the idea was born during the procedure. The case was also removed from analysis when any of the records prevented vessel assessment before or after stenting due to poor quality. Such situations comprised, e.g., insufficient contrast washout resulting in the inability to calculate vessel lumen or EEL before surgery as well as stent expansion following the procedure. For this reason, few patients were removed from the analysis, which constituted less than 10% of all patients participating in the pre-analysis of the current study. In the case of satisfactory recordings, the analysis of OCT images was performed by at least 1 or 2 highly experienced operators during the procedure. Blinded analysis was also carried out by an experienced and blinded operator in the evaluation of OCT images, who finally analyzed all cases before entering the database.

Adding diagnostic ability of plaque characteristic and easier planning of stenting strategy using artificial intelligence are among the main innovations of Ultreon™ 1.0 software. In the current analysis, prior to PCI, we assessed plaque type, maximal calcium angle, maximal calcium thickness and length, minimal lumen diameter (MLD), distal reference EEL diameter and distal lumen diameter (in the case of lacking EEL). After PCI, we looked for medial dissection, malapposition, minimum stent expansion and MLD. Then, we additionally calculated the distal reference EEL diameter to minimum stent diameter, distal lumen diameter (in the case of no EEL) to minimum stent diameter and MLD after PCI to minimum stent diameter ratios.

### 2.3. Minimal Mean Stent Expansion

The main parameter assessed in the present publication related to OCT use comprised minimal mean stent expansion assessed automatically by Ultreon™ 1.0 Software. In the majority of cases, the minimal stent expansion diameter was assessed via Ultreon™ 1.0 Software. However, in each instance, the result was manually checked and adjusted. If necessary, this was even performed frame-by-frame. Incorrect measurements, e.g., insufficient contrast washout or far deviation from the oval cross-section of the artery, were corrected manually and replaced with the appropriate result. This was performed because such situations can often cause problems with the correct tracing of the artery lumen by the software.

### 2.4. Study Endpoints

The first parameter subjected to the comparative assessment was the measurement of minimal mean stent expansion after PCI. The next indexes subjected to comparative analysis were the clinical characteristics of the PCI procedure indices as well as other parameters assessed by OCT, such as the incidence of medial dissections.

Other study endpoints of the current analysis included assessment of the frequency of MACEs occurrence during follow-up period. These were considered as the following: cardiac death, myocardial infarction (MI), revascularization (either surgical (CABG, coronary artery bypass grafting) or percutaneous (Re-PCI; TLR, target lesion revascularization; TVR, target vessel revascularization)) and/or cerebrovascular events, e.g., stroke or transient ischemic events. Device-oriented composite endpoints, including cardiac death, target vessel related myocardial infarction (TV-MI) as well as target lesion revascularization (TLR), were also evaluated.

### 2.5. Statistical Analysis

Categorical variables are given as numbers and percentages. Continuous variables are demonstrated as means ± standard deviations, and additionally, as medians and interquartile ranges in the case of non-normal data distribution. Normality of distribution was analyzed with the Shapiro-Wilk test [15]. Equality of variance was subjected to evaluation by implementing Levene’s test. The differences noted between groups were subject to comparison via the Student’s or Welch’s *t*-tests. This was dependent on the equality of variance regarding normally distributed variables. The Mann-Whitney U test was used for continuous variables lacking normal distribution. Categorical variables were also compared via Pearson’s chi-squared or Fisher’s exact test. This was conducted when 20% of cells had a count below 5. Ordinal variables were further compared via the Cochran-Armitage trend test. We drew Kaplan-Meier curves in order to analyze the survival rate in designated risk groups. Wanting to test outcome differences in the groups, the log-rank statistic was implemented.

All baseline, demographic and procedural characteristics were assumed as possible predictors of stent expansion in univariable linear mixed effect models. The clustering effect of multiple stents/procedures per single patients treated as random effects was considered. Then, variables with a *p*-value < 0.2 or those of supposed significance were added to the multivariable model.

Final multivariable regression models were designed by minimizing Akaike Information Criterion to discover the predictors of stent expansion in the U and non-U software groups. All of the performed statistical analyses were carried out using JMP^®^, Version 13.1.0 (SAS Institute INC., Cary, NC, USA) and R 4.1.1 (R Foundation for Statistical Computing, Vienna, Austria, 2021) with the ‘lme4’ package, version 1.1-27.1.

## 3. Results

### 3.1. General Characteristics and Concomitant Disease at Baseline (Before Index Procedure)

Between the U and non-U groups, no statistically significant differences were noted with regard to general characteristics or concomitant diseases (Table 1).

### 3.2. Biochemical Indices at Baseline (Before Index Procedure)

Considering selected biochemical indices, no statistically significant differences were observed between the studied groups of patients. The following indices were taken into account, e.g., kidney function indices, blood cell counts, markers of myocardial injury and proinflammatory markers (Table S1).

### 3.3. Procedural Indices (Index Procedure)

Patients representing the U group were more frequently subjected to treatment using drug-eluting stent (DES) implantation. Bioresorbable scaffolds (BRS) were implemented less frequently compared to the non-U software group (Table 2).

### 3.4. OCT Parameters (Index Procedure)

The acquired images were transferred and processing via non-U software for the novel software assessment. It was found that the mean distal reference EEL diameter was significantly greater when compared to initial assessment by Ultreon™ 1.0 Software (3.72 ± 0.82 vs. 3.25 ± 0.7 mm, *p* = 0.02). Additionally, distal EEL reference diameter to minimum stent diameter ratio was greater in the non-U software group when compared to U (1.26 ± 0.25 vs. 1.08 ± 0.15 mm, *p* = 0.003) (Table 3).

### 3.5. Pharmacotherapy (After Index Procedure)

With regard to medical treatment following discharge from hospital, no statistically significant between-group differences were noted among the studied patient groups (Table S2).

### 3.6. Clinical Outcomes

The mean follow-up duration lasted longer in the non-U software group in comparison to the U (82.5 ± 76.6 vs. 485.7 ± 470 days, *p* < 0.001). Total completeness of follow-up was 98.9% (98.2% in the U and 100% in the non-U group). All three TLRs occurred in the non-U group (*p* = 0.01). All of these cases were of in-stent restenosis (no-thrombosis), while all six TVRs occurred within the non-U group (*p* = 0.001). Furthermore, myocardial infarctions, MACEs and re-PCIs took place more commonly in the non-U group (Table 4).

**Figure 1:**
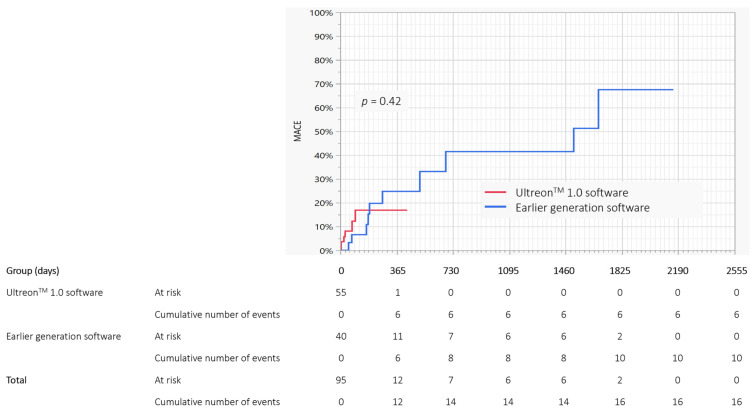
Comparison of Kaplan-Meier survival estimates according to the MACE occurrence in the Ultreon™ 1.0 and earlier generation software group. MACE, major adverse cardiovascular events.

When assessing Kaplan-Meier estimates, there were no differences in free survival from TLR (*p* = 0.65), TVR (*p* = 0.53), MI (*p* = 0.34), stroke (*p* = 0.32), MACE (*p* = 0.42 (Figure 1) or cardiac death (*p* = 0.13). Due to the fact that all deaths occurred in the U group, the difference between both estimated groups was also confirmed by Kaplan-Meier estimates (*p* = 0.04). Furthermore, because the number of events was low, it was not possible to estimate the *p*-value for TV-MI by Kaplan-Meier estimates.

### 3.7. Predictors of Stent Expansion—Univariable Analysis

Among the factors significantly related to better stent expansion assessed by univariable analysis, we found lack of chronic obstructive pulmonary disease and bronchial asthma, greater minimal stent diameter, lack of treatment with inhalators (glucocorticosteroids/bronchodilatators) and greater blood hemoglobin levels. In contrast, higher age, higher Euroscore II value, STS and SYNTAX scores for 4-year survival following CABG were noted among the factors related to poorer stent expansion (Table S3).

### 3.8. Predictors of Stent Expansion—Multivariable Analysis

A family history of cardiovascular diseases and sirolimus antiproliferative agents were found to be significantly correlated with poorer stent expansion assessed by multivariable analysis (Table 5).

## 4. Discussion

Among the main findings of the current study, it was noted that there were not any significant differences concerning general characteristics between either of the assessed groups at baseline.

Secondly, in the era of earlier generation software used for processing OCT images, this imaging technique was used in a substantial percentage of patients for PCI with BRS stenting. In contrast, nowadays, it is mostly applied in DES stenting. Moreover, PCIs in the group of non-U software for OCT were more frequently related to PCI without stent implantation or single-stent implantation. In comparison, U software was more frequently used in complex procedures.

Thirdly, the distal reference EEL diameter was significantly greater in the non-U group following assessment using the U software. This was also visible for the distal reference to minimal stent diameter ratio, which was greater in the earlier generation software group. This was found apart from the fact that there was a lack of significant differences with regard to minimal or maximal stent diameter between the study groups.

Fourthly, the period of follow-up, by design, was greatly longer for the non-U software group. Selected study endpoints also occurred more often in the earlier generation software, including TLRs, with all in-stent restenosis, TVRs, myocardial infarctions, re-PCIs and MACEs. The only contrasting significant difference was noted for all-cause mortality due to the fact that all deaths occurred in the U group.

### 4.1. Predictors of Stent Expansion

Considering the predictors of stent expansion, univariable analysis allowed to demonstrate a significant relationship between the extent of stent expansion and COPD/bronchial asthma, mean minimal stent diameter, treatment with inhalators for CAPD/bronchial asthma, age, Euroscore II, STS and SYNTAX Score II for 4-year survival following CABG and blood hemoglobin concentration evaluation. However, multivariable analysis allowed to confirm a relationship between family history of cardiovascular diseases and the sirolimus antiproliferative agent. Neither univariable nor multivariable analysis showed a correlation between the type of software used for OCT image acquisition and extent of stent expansion.

In previously published studies, it has been demonstrated that the maximum arc of target lesion calcification is a predictor of stent expansion [16,17,18,19]. This is also a strong predictor regarding outcomes following the implantation of drug-eluting stents [17,18,19]. OCT is the preferred imaging modality when wanting to detect the calcification occurrence and intensity. This is because, in contrast to angiography or IVUS, OCT can differentiate deep from superficial calcium. At the same time, it can measure calcium thickness, which is a strong predictor of stent under-expansion [20]. Such complex evaluation concerning calcific plaque before PCI can aid the implementation of a strategy needed to prepare the vessel and determine the efficacy of the applied treatment [21,22]. In several recent studies, calcium fracture following lesion preparation has been exhibited as a predictor of stent expansion. This was established using OCT [23,24,25].

In the current study, we did not assess the presence of calcium rupture after lesion preparation or before stent implantation. However, we did not observe a relationship between stent expansion and calcifications in terms of arc, thickness or length. We conclude that this effect could be decreased in the current analysis due to there being 50% of patients with confirmed severe calcifications by OCT in the overall group of patients (both groups). What is more, almost half of them were treated with rotablation, while nearly a quarter of them with IVL before stent implantation.

As previously published, some circumstances may impede the identification of the EEL, such as reach with lipid or calcium plaques [26]. Additionally, the selection of stent, in particular, may have had impact in the past. Thus, in the case of BRS-type stents, less calcified lesions were subjected to angioplasty, inter alia, in order to avoid damage to the stent during deployment and post-dilatation. This was additionally performed to improve follow-up assessment and outcomes.

Based on the results of the current study, in such cases, the diameter of the vessel lumen was more often considered than the EEL when selecting the stent. However, similar practices were introduced for other stent types. Such a view was mainly related to non-U software, since U software is available, BRS stents are practically no longer obtainable. Moreover, attitudes towards stent measures before deployment have changed, which was mainly based on studies in which pre-PCI IVUS and OCT were compared for stent diameter selection [7]. Another issue is that distal EEL diameter, even with the use of modern software, may be visualized via OCT among 85% of the cases by the particular catheterization laboratory. In 95% of cases, this can be conducted by the OCT core laboratory within 5 mm of the original angiographically selected segment for reference [27].

A subsequent issue is the presence of fibroatheromatic plaque. It has been demonstrated that stent edge implantation within a fibroatheroma cap could rise edge issues in the case of both stent thrombosis and MACE [17,28,29,30,31,32,33]. Similar consequences could be adjusted for heavily calcified lesions, even after prior modification with dedicated devices (rotational and orbital atherectomy, lithotripsy or laser atherectomy). In the past, the majority of operators used OCT luminal dimensions for the sizing of guide stents, which caused poorer results than those achieved using EEL- or midwall-based guidance by IVUS [1,2,3]. It has been proved that an EEL-based strategy of stent sizing caused the minimal stent area to be similar to that in the case of IVUS-guided PCI. At the same time, a lower number of untreated post-PCI complications was observed [27].

### 4.2. Follow-Up

Due to the large disproportion in the number of deaths between both groups of patients, also in light of a much shorter follow-up period, we analyzed each case of death in the U group. Based on this analysis, there was one 61-year-old male with LVEF 25%, PCI-assisted with the Impella pump and pre-procedural SYNTAX score II for 4-year mortality in the case of percutaneous treatment equal to 31%. This patient died 6 days following the procedure. The second patient was a 64-year-old male, with LVEF 20%, no LVAD and pre-procedural SYNTAX score II for 4-year mortality in the case of percutaneous treatment equal to 47%. This patient also died 6 days post-procedure. The third patient was a 79-year-old male with LVEF 18%, PCI assisted with the Impella pump and pre-procedural SYNTAX score II for 4-year mortality in the case of percutaneous treatment equal to 44%. This patient died 24 days following the procedure. The fourth male patient, who was 64 years old, with LVEF 25%, PCI assisted with the Impella pump and pre-procedural SYNTAX score II for 4-year mortality in the case of percutaneous treatment equal to 49%, died 39 days after the procedure. The fifth patient was a 92-year-old male, with LVEF 45% and pre-procedural SYNTAX score II for 4-year mortality in the case of percutaneous treatment equal to 15%. He died 77 days post-procedure. In summary, these patients were at a significantly increased death risk rate. They would have been disqualified from any surgical treatment at many centers.

It is also difficult to comment on the comparison in MACE frequency, and the fact that the difference was mainly due to TVR and TLR because the average length of the follow-up period was relatively short in the U group. Therefore, in order to draw more reliable conclusions, a longer follow-up period is recommended. The size of the patient group should also be significantly increased. Another problem that significantly limits the interpretation of the TLR frequency in the current analysis is the different characteristics of patients and stents, i.e., a kind of generation gap.

### 4.3. Issues Related to the Procedure and Stent Expansion

In the literature on the subject, authors have provided various criteria related to stent expansion conducted in an optimal manner [34]. In some studies, an in-stent minimal lumen area larger than or equal to 80% of the average reference with symmetric stent expansion was sought [35]. The criteria for other trials required the minimal stent region to be greater than or equal to the distal reference lumen area [36].

In ILUMIEN III, the segment subjected to stenting was divided in half to achieve a minimal stent area in the proximal and distal parts of at least 90% concerning the respective reference segment lumen area [7]. The latter recommendations seem to dominate nowadays, and they are mostly spread among interventional cardiologists.

According to current guidelines, it is advised to carry out post-dilation with a non-compliant balloon adjusted to the respective reference EEL diameter. On the basis of the findings from the present study, a large disproportion between maximal stent diameter and the balloon used for post-dilatation is related to the proximal optimization technique. We are not able to compare this with the proximal reference EEL diameter because we concentrated on the distal one. Therefore, we do not possess data for the proximal EEL reference diameter. On the other hand, the distal half of the stent post-dilatation was difficult to unravel. This was due to the fact that, in several cases, balloons larger in diameter were used for post-dilatation of the distal edge compared to the distal EEL reference diameter at lower pressures. This was caused by several issues, including economic considerations. Proper stent tension reduces the likelihood of malapposition, which could be related to increased stent thrombosis. However, such relationships have not been proven in all studies [37,38,39,40].

Acute findings following stenting, which include edge dissection, intramural hematoma, tissue protrusion and incomplete stent apposition, are noticeable in OCT with a greater sensitivity than compared to IVUS [7]. Edge dissections occurred in the current study in one case in the U-group and one in the non-U group. For calculations, we only selected those edge dissections that demanded, according to current recommendations, additional stent placement. It can additionally be concluded that the increase in the size of selected stents for the EEL reference diameter compared to the lumen reference diameter does not have direct impact on the frequency of marginal medial dissections. Nonetheless, such impact is noted in post-dilation with EEL balloons at high pressures. The features of dissection that require stenting have been described in previous research [2,32,41].

## 5. Conclusions

The most significant finding of the current study is that improvement in the software for image acquisition and processing was not related to the mean extent of stent expansion after the percutaneous intervention. We conducted an evaluation in selected indices concerning the procedure, including stent types and number, which indirectly translates into procedure complexity. Analyzing the stent selection technique itself, the comparison of the distal EEL diameter reference parameters before PCI indicates that, in the case of the latest version of the OCT processing software, the EEL diameter is mainly used to select the distal stent diameter.

## 6. Limitations

Undoubtedly, the present results are preliminary. The study group was limited to a small size, and strong conclusions cannot be drawn. In addition, by observing the patients included in the study, we found certain bias in the selection of patients. For example, those from the Impella group were characterized by significantly lower left ventricular ejection fraction. They demonstrated a highly significant relationship with long-term prognosis, despite the fact that the estimated peri-procedural mortality risk was similar in both groups. The presence of a learning curve among individual operators is also of great importance. The initial results of the first treatments may differ significantly from subsequent, systematized treatments.

Due to the small group of patients, they were not matched using propensity score matching. Such a procedure would take a number of features, including patient age, concomitant diseases, vessel diameter, degree of calcification, or the urgency of the procedure, into account. This is a substantial limitation of the study. In its the current form, this may be considered a pilot study.

The Ultreon 1.0™ software enables the performance of more complicated procedures in the more burdened patients who, in many cases, would be qualified for conservative treatment. Artificial intelligence, aiding the improvement of percutaneous interventions, contributes to shortening procedure duration and enhancing its effects.

However, proving the superiority of the latest software over the previous ones and translating them into specific numbers and results, is difficult. This is because the characteristics of patients who undergo OCT intravascular imaging are constantly evolving. It is extremely difficult to collect a large group of patients in a short time to obtain reliable results, at least in the current stage of OCT prevalence. Additionally, this is despite the fact that it is increasing exponential in Poland, if only due to the fact that the costs of its use have been reimbursed by the National Health Fund since January 2022.

The results are also influenced by the presence of previous BRS stent software in the prior software group, which may undoubtedly modify the results. This could, for example, be related to a higher thrombosis rate. On the other hand, this may be offset by a less complex treatment.

The comparative analysis of both analyzed groups in terms of the duration of the procedure was deliberately omitted due to the fact that patients were not selected. Recently, OCT has been used in many cases for very complex treatments in multivessel disease with the use of devices supporting the work of the left ventricle as well as plaque debulking devices. The duration of these procedures, as well as the exposure to radiation and the dose of contrast, were relatively high compared to the procedures performed in previous years, where OCT was more often used for PCI with implantation of one stent into one vessel. It can be presumed with high probability that, in the case of patient selection in terms of the complexity of PCI procedures, the contrast dose and radiation exposure would be lower with the use of the latest software. However, in the case of the duration of the procedure, the latest software certainly makes the procedure smoother for the operator, and probably also faster due to the use of some kind of artificial intelligence. We intend to make such comparisons after gathering more patients.

## Figures and Tables

**Table 1:** General characteristics and concomitant diseases.

	Total N = 95	Ulteron™ 1.0 Software N = 55	Earlier Generation Software N = 40	*p*-Value
Age, years	66.1 ± 12.9 67.7 (61.3; 75.1)	68.4 ± 10.8 68.3 (62.4; 75.8)	63 ± 14.9 65.8 (57.8; 70.6)	0.07
Gender, male	74 (77.9)	44 (80)	30 (75)	0.62
Euroscore II, %	3 ± 2.5 2.1 (1.2; 4.1)	2.9 ± 2.3 2 (1.2; 4.2)	3.2 ± 2.7 2.3 (1.4; 3.7)	0.58
STS score	2 ± 1.8 1.5 (0.7; 2.8)	2 ± 1.8 1.3 (0.6; 2.8)	2.1 ± 1.7 1.6 (0.7; 3.3)	0.76
Syntax I	15.2 ± 9.2 13 (8; 21)	16 ± 9.4 13.5 (8; 24)	14.2 ± 9.1 13 (7.2; 19)	0.35
Syntax II PCI 4-year mortality, %	18.2 ± 18 10 (5.3; 23.9)	19.2 ± 18.2 13.9 (5.9; 23.6)	16.9 ± 17.8 9.2 (4.2; 27.6)	0.54
Syntax II CABG 4-year mortality, %	12 ± 10.4 9.1 (4.1; 17.2)	12.3 ± 10.1 9.1 (4.8; 17.4)	11.7 ± 11.1 8.5 (3.1; 16.8)	0.4
Diabetes mellitus	33 (34.7)	22 (40)	11 (27.5)	0.27
Hypercholesterolemia	56 (58.9)	29 (52.7)	27 (67.5)	0.2
Arterial hypertension	79 (83.2)	46 (83.6)	33 (82.5)	0.88
Kidney failure	7 (7.4)	5 (9.1)	2 (5)	0.69
Dialysotherapy	1 (1)	1 (1.8)	0 (0)	0.3
Prior PCI	58 (61)	36 (65.4)	22 (55)	0.39
Prior CABG	6 (6.3)	1 (1.8)	5 (12.5)	0.95
COPD/Bronchial asthma	14 (14.7)	8 (14.5)	6 (15)	0.95
Smoking	26 (27.4)	14 (25.4)	12 (30)	0.79
Family history of CVD	14 (14.7)	5 (9.1)	9 (22.5)	0.08
Heart failure	47 (49.5)	29 (52.7)	18 (45)	0.53
LVEF, %	42 ± 16.4 45 (29.5; 55)	40.5 ± 16 42.5 (28.5; 55)	44 ± 16.9 47 (32.5; 55)	0.33
Peripheral artery disease	13 (13.7)	5 (9.1)	8 (20)	0.14
Prior PTCA	3 (3.2)	1 (1.8)	2 (5)	0.57
Prior stroke	7 (7.4)	5 (9.1)	2 (5)	0.69

Data are expressed as mean ± standard deviation and median ÷ interquartile range or numbers (percentages). CABG, coronary artery bypass grafting; COPD, chronic obstructive pulmonary disease; CVD, cardiovascular disease; LVEF, left ventricle ejection fraction; PCI, percutaneous coronary intervention; STS, Society of Thoracic Surgeons.

**Table 2:** Procedural indices.

	Total N = 95	Ulteron™ 1.0 Software N = 55	Earlier Generation Software N = 40	*p*-Value
LMCA	27 (28.4)	17 (30.9)	10 (25)	0.52
LAD	69 (72.6)	43 (78.2)	26 (65)	0.15
Diagonal branch	13 (13.7)	11 (20)	2 (5)	0.03
Circumflex branch	27 (28.4)	15 (27.3)	12 (30)	0.77
Marginal branch	5 (5.3)	2 (3.6)	3 (7.5)	0.64
Right coronary artery	30 (31.6)	15 (27.3)	15 (37.5)	0.28
Chronic total occlusion	8 (8.4)	5 (9.1)	3 (7.5)	1.0
PCI + stent	87 (91.6)	52 (94.5)	35 (87.5)	0.27
Drug-eluting balloon	4 (4.2)	2 (3.6)	2 (5)	1.0
Drug-eluting stent	82 (86.3)	52 (94.5)	30 (75)	0.006
Bioresorbable scaffold	6 (6.3)	0 (0)	6 (15)	0.004
Type of antimitotic agent:				
Everolimus	72 (76.6)	45 (81.8)	27 (69.2)	0.17
Sirolimus	18 (18.9)	7 (12.7)	11 (27.5)	0.07
Zotarolimus	2 (2.1)	2 (3.6)	0 (0)	0.5
Number of stents	1.8 ± 1.1 2 (1; 2)	1.9 ± 1.1 2 (1; 2)	1.7 ± 1.2 1 (1; 3)	0.22
Number of stents:				0.03
0	8 (8.4)	3 (5.4)	5 (12.5)	
1	35 (36.8)	17 (30.9)	18 (45)	
2	30 (31.6)	24 (43.6)	6 (15)	
3	14 (14.7)	7 (12.7)	7 (17.5)	
4	6 (6.3)	2 (3.6)	4 (10)	
5	2 (2.1)	2 (3.6)	0 (0)	
Total stent length, mm	46.1 ± 28.6 41 (24; 66)	48.8 ± 25.2 48 (28; 66)	42.4 ± 32.7 38 (18; 72.7)	0.12
Maximum stent diameter, mm	3.4 ± 3.2 3.5 (3; 3.5)	3.7 ± 4.1 3.5 (3; 3.5)	2.9 ± 1.2 3.5 (3; 3.5)	0.31
Minimum stent diameter, mm	2.7 ± 1 3 (2.5; 3.5)	2.8 ± 0.9 3 (2.5; 3.5)	2.6 ± 1.1 2.6 (2.5; 3.4)	0.27
Maximum balloon diameter, mm	4.1 ± 0.9 4 (3.5; 5)	4.1 ± 1 4 (3.5; 5)	4.1 ± 0.9 3.7 (3.5; 4.5)	0.7
Maximum balloon pressure, atm.	21.4 ± 6.3 20 (18; 24)	22.1 ± 6.9 20 (18; 25)	20.6 ± 5.3 20 (16; 24)	0.19
Rotablation	22 (23.2)	14 (25.4)	8 (20)	0.53
IVL	8 (8.4)	5 (9.1)	3 (7.5)	1.0
Impella pump	25 (26.3)	14 (25.4)	11 (27.5)	0.82
IABP	3 (3.2)	1 (1.8)	2 (5)	0.57

Data are expressed as mean ± standard deviation and median ÷ interquartile range where necessary (non-normal distribution) or numbers (percentages). IABP, intra-aortic balloon pump; IVL, intravascular lithotripsy; LAD, left anterior descending artery; LMCA, left main coronary artery; PCI, percutaneous coronary intervention.

**Table 3:** Optical coherence tomography parameters.

	Total N = 95	Ulteron™ 1.0 Software N = 55	Earlier Generation Software N = 40	*p*-Value
Before PCI				
Type of plaque:				0.63
- Lipidic	8 (9.4)	5 (9.1)	3 (10)	
- Fibrotic	14 (16.5)	8 (14.5)	6 (20)	
- Mild/moderate calcium	21 (24.7)	12 (21.8)	9 (30)	
- Severe calcium	42 (49.4)	30 (54.5)	12 (40)	
Maximum calcium angle, °	170.2 ± 122.7 181 (0; 289)	174.7 ± 121.8 183 (59; 296)	162.2 ± 125.9 180 (0; 261)	0.71
Maximum calcium thickness, mm	0.9 ± 0.6 1.1 (0; 1.4)	0.9 ± 0.6 1.1 (0.5; 1.4)	0.9 ± 0.7 1 (0; 1.3)	0.51
Total calcium length, mm	11 ± 13.7 5 (0; 19)	11.6 ± 13.3 5 (2; 20)	9.9 ± 14.5 3 (0; 13.5)	0.27
Minimum lumen diameter, mm	1.5 ± 0.44 1.4 (1.2; 1.7)	1.46 ± 0.4 1.38 (1.1; 1.6)	1.6 ± 0.5 1.52(1.2; 2)	0.18
Distal EEL reference diameter, mm	3.39 ± 0.76 3.26 (2.9; 3.8)	3.25 ± 0.7 3 (2.84; 3.75)	3.72 ± 0.82 3.56 (3.2; 4.1)	0.02
Distal EEL reference to minimum stent diameter ratio	1.13 ± 0.2 1.09 (1.01; 1.2)	1.08 ± 0.15 1.08 (1; 1.14)	1.26 ± 0.25 1.2 (1.1; 1.34)	0.003
Distal lumen diameter, mm	2.56 ± 0.8 2.43 (2; 2.95)	2.5 ± 0.74 2.36 (1.94; 2.94)	2.67 ± 0.82 2.7 (2.1; 3.2)	0.31
Distal lumen to minimum stent diameter ratio	0.84 ± 0.2 0.82 (0.7; 0.94)	0.82 ± 0.2 0.8 (0.7; 0.93)	0.9 ± 0.25 0.9 (0.7; 1)	0.1
After PCI				
Medial dissection	2 (2.35)	1 (1.82)	1 (3.33)	0.66
Malapposition	1 (1.18)	1 (1.82)	0 (0)	1.0
Minimum stent expansion, %	96 ± 16.7 95 (86; 106)	96.2 ± 16.2 94 (86.5; 105)	95.8 ± 17.9 96.5 (85; 108)	0.91
Minimum lumen diameter, mm	2.7 ± 0.6 2.7 (2.3; 3.1)	2.64 ± 0.6 2.6 (2.2; 3)	2.8 ± 0.6 2.7 (2.4; 3.2)	0.32
Minimum lumen to minimum stent diameter ratio	0.92 ± 0.16 0.9 (0.84; 0.97)	0.89 ± 0.14 0.89 (0.82; 0.97)	0.96 ± 0.18 0.94 (0.9; 1)	0.06

Data are expressed as mean ± standard deviation and median ÷ interquartile range where necessary (non-normal distribution) or numbers (percentages). EEL, external elastic lamina; PCI, percutaneous coronary intervention.

**Table 4:** Clinical outcomes.

	Total N = 95	Ulteron™ 1.0 Software N = 55	Earlier Generation Software N = 40	*p*-Value
Completed follow-up	94 (98.9)	54 (98.2)	40 (100)	1.0
TLR:	3 (3.5)	0 (0)	3 (9.4)	0.01
- In-stent restenosis	3 (100)	0 (0)	3 (100)	
- In-stent thrombosis	-	-	-	
TVR	6 (7.1)	0 (0)	6 (18.7)	0.001
Myocardial infarction	2 (2.3)	0 (0)	2 (6.2)	0.04
Stroke	2 (2.3)	1 (1.9)	1 (3.1)	0.71
Re-PCI	6 (7.1)	1 (1.9)	5 (15.6)	0.02
CABG	0 (0)	0 (0)	0 (0)	-
Cardiac death	3 (3.5)	3 (5.6)	0 (0)	0.09
TV-MI	1 (1.2)	0 (0)	1 (3.1)	0.16
MACE	16 (18.4)	6 (10.9)	10 (31.2)	0.01
Death overall	5 (5.7)	5 (9.1)	0 (0)	0.03
Mean follow-up duration, days	252.3 ± 479.2 98 (31; 196)	82.5 ± 76.6 56 (26; 126)	485.7 ± 670 189 (67; 467)	<0.001

Data are expressed as mean ± standard deviation and median ÷ interquartile range where necessary (non-normal distribution) or numbers (percentages). CABG, coronary artery bypass grafting; MACE, major adverse cardiac events; Re-PCI, repeat percutaneous coronary intervention; TLR, target lesion revascularization; TVR, target vessel revascularization.

**Table 5:** Predictors of stent expansion—multivariable analysis.

Variable	Estimate	95% Confidence Interval	*p*-Value
Ultreon 1.0 vs. older software	2.29	−4.207–8.788	0.5
History of CVD, yes vs. no	−13.387	−22.722–(−4.052)	0.008
Sirolimus, yes vs. no	−10.893	−19.226–(−2.56)	0.01
Insulinotherapy, yes vs. no	8.299	0.369–16.228	0.053
Inhalators, yes vs. no	−10.93	−21.896–0.035	0.06
Age, years	−0.206	−0.462–0.05	0.13
Hemoglobin, g/dL	1.904	0.026–3.783	0.06
Maximum stent length, mm	−0.851	−1.781–0.078	0.09

CVD, cardiovascular disease.

## Data Availability

Upon special request.

## Supplementary Materials

The following supporting information can be downloaded at: https://www.mdpi.com/article/10.3390/jcdd9070218/s1, Table S1: Biochemical indices; Table S2: Pharmacotherapy; Table S3: Predictors of stent expansion—univariable analysis.
