# Supplementary material for: Procedure-Related Differences and Clinical Outcomes in Patients Treated with Percutaneous Coronary Intervention Assisted by Optical Coherence Tomography between New and Earlier Generation Software (Ultreon™ 1.0 Software vs. AptiVue™ Software)
Source: J Cardiovasc Dev Dis. 2022 Jul 6;9(7):218. doi: 10.3390/jcdd9070218 (PMC9318712; doi:10.3390/jcdd9070218)
Supplement: Supplementary file 1 [file jcdd-09-00218-s001.zip › jcdd-1799648-supplementary.pdf]

Table S1. Biochemical indices.

	Total N = 95	Ulteron™ 1.0 software N = 55	Earlier generation software N = 40	*P*-value
Creatinine before PCI	91 ± 23.9<br>88.5 (73.5; 104.2)	93.2 ± 24.3<br>90 (75.7; 106.5)	87.8 ± 23.3<br>82.6 (71.9; 100.6)	0.24
Creatinine after PCI	95.8 ± 29.5<br>89 (74; 111)	94.2 ± 24.4<br>89.2 (77; 109.2)	98.1 ± 35.7<br>87.5 (71.3; 123)	0.56
GFR, mL/min.	78.6 ± 26.8<br>76 (63; 85)	76 ± 28.9<br>73 (58; 83)	82.1 ± 23.4<br>78 (66; 96.5)	0.27
PLT, $*10^3/\mu L$	248.2 ± 78.8<br>234 (191; 290)	251.7 ± 81.6<br>240 (180; 318)	243.3 ± 75.4<br>232.5 (196.5; 260.7)	0.66
Haemoglobin before PCI, mg/dL	13.7 ± 1.8<br>13.6 (13; 14.7)	13.6 ± 1.7<br>13.5 (12.9; 14.5)	13.9± 1.9<br>13.7 (13; 15)	0.32
Haemoglobin after PCI, mg/dL	12.5 ± 2.7<br>12.8 (10.9; 13.9)	12.6 ± 2.2<br>12.8 (10.9; 14.1)	12.2 ± 3.3<br>12.8 (10.9; 13.4)	0.95
RBC, $*10^6/\mu L$	4.5 ± 0.5	4.4 ± 0.5	4.6 ± 0.6	0.07
CRP, mg/dL	17.1 ± 33.8<br>10.6 (2.4; 19.7)	14.7 ± 11<br>11.3 (5.3; 23.8)	21.1 ± 53.9<br>2.5 (1.2; 17)	0.09
Total cholesterol, mmol/L	4 ± 1.4<br>3.7 (3; 4.7)	4.1 ± 1.4<br>3.8 (3.2; 4.8)	3.9 ± 1.3<br>3.6 (2.8; 4.8)	0.45
LDL cholesterol, mmol/L	2.1 ± 1.2<br>1.80 (1.38; 2.70)	2.2 ± 1.2<br>1.90 (1.40; 2.70)	2.1 ± 1.2<br>1.80 (1.10; 2.85)	0.6
TGL, mmol/L	1.5 ± 0.7<br>1.4 (1; 1.8)	1.5 ± 0.6<br>1.4 (1; 1.8)	1.6 ± 0.9<br>1.3 (1; 1.9)	0.81
HDL cholesterol, mmol/L	1.2 ± 0.4<br>1.1 (0.9; 1.3)	1.2 ± 0.4<br>1.1 (0.9; 1.3)	1.1 ± 0.3<br>1 (0.9; 1.3)	0.79
Maximal CK-Mb, IU/L	60.9 ± 81.7<br>24 (16; 74.7)	50.8 ± 78.8<br>22.5 (15.7; 46.3)	74.7 ± 85.4<br>26 (18.2; 112)	0.23
Maximal troponin T, pg/mL	2943.6 ± 7341.8<br>329 (65.1; 1307)	1084.3 ±1517.2<br>426 (121; 1305)	5303.5 ± 10571.2<br>284 (24.46; 4534)	0.58
NT-pro BNP, pg/mL	2807.5 ± 3926.7<br>1592 (805; 3013)	2897.3 ± 4439.9<br>1899 (504; 3017)	2578 ± 2335.9<br>1511 (1136.5; 4158)	0.6
Glucose, mmol/L	6.9 ± 2.1<br>6.1 (5.3; 8.1)	7.2 ± 2.2<br>6.2 (5.3; 8.5)	6.3 ±1.8<br>5.8 (5.3; 6.5)	0.31

Data are expressed as mean ± standard deviation and median ÷ interquartile range where necessary (non-normal distribution) or numbers (percentages). BNP, B-type natriuretic peptide; CK-Mb, creatine kinase myocardial bound; CRP, C-reactive protein; GFR, glomerular filtration rate;

HDL, high-density lipoproteins; LDL, low-density lipoproteins; NT, N-terminal; PCI, percutaneous coronary intervention; PLT, platelet count; RBC, red blood cells; TGL, triglycerides.

Table S2. Pharmacotherapy.

	**Total N = 95**	**Ulteron™ 1.0 Software N = 55**	**Earlier generation software N = 40**	***P*-value**
Acetyl-salicylic acid	91 (95.8)	52 (94.5)	39 (97.5)	0.46
P_2Y_{12} inhibitor:				
- clopidogrel	50 (52.6)	29 (52.7)	21 (52.5)	0.98
- prasugrel	10 (10.5)	6 (10.9)	4 (10)	0.88
- ticagrelor	33 (34.7)	19 (34.5)	14 (35)	0.96
Antithrombotic treatment (heparins, old and new oral anticoagulants)	16 (16.8)	9 (16.4)	7 (17.5)	0.88
Lipid-lowering treatment				
- statin	89 (93.7)	51 (92.7)	38 (95)	0.64
- fibrate	3 (3.16)	1 (1.82)	2 (5)	0.38
- ezetimibe	10 (10.5)	8 (14.5)	2 (5)	0.11
Insulinotherapy	14 (14.7)	9 (16.4)	5 (12.5)	0.59
Oral anti-diabetic therapy	22 (23.2)	16 (29.1)	6 (15)	0.1
Angiotensin converting enzyme inhibitors	80 (84.2)	45 (81.8)	35 (87.5)	0.44
Calcium channel blockers	25 (26.3)	13 (23.6)	12 (30)	0.48
Beta blocker	85 (89.5)	49 (89.1)	36 (90)	0.89
Inhalators (bronchodilatators/GCSs)	10 (10.5)	4 (7.3)	6 (15)	0.23

Data are expressed as mean ± standard deviation and median ÷ interquartile range where necessary (non-normal distribution) or numbers (percentages). GCSs, glicocorticosteroids.

Table S3. Predictors of stent expansion – univariable analysis.

Variable	Estimate	95% Confidence Interval	*P*-value
Ultreon 1.0 vs. older software	0.407	(−3.182)–3.998	0.82
COPD/bronchial asthma, no vs. yes	6.367	2.103–10.631	0.004
Minimal stent diameter, mm	5.091	0.968–9.214	0.016
Inhalators (GKSs/bronchodilatators) , no vs. yes	6.795	1.638–11.952	0.01
Age, years	−0.313	(−0.591)–(−0.035)	0.03
Euroscore II, %	−1.409	(−2.787)–(−0.032)	0.045
STS score, %	−2.044	(−4.007)–(−0.082)	0.041
SYNTAX score II, 4 years survival for CABG	−0.41	(−0.749)–(−0.071)	0.018
Haemoglobin, mg/dL	2.558	0.716–4.399	0.007
Diabetes mellitus, no vs. yes	−2.599	−6.126–0.927	0.15
Dialysotherapy, no vs. yes	12.595	−3.956–29.148	0.13
Prior PCI, no vs. yes	2.51	−0.902–5.924	0.14
History of CVD, no vs. yes	4.482	−0.781–9.745	0.09
Prior peripheral percutaneous interventions, no vs. yes	6.846	−2.829–16.521	0.16
Sirolimus eluting stent, no vs. yes	3.413	−1.101–7.927	0.14
Total length of stents, mm	0.112	−0.013–0.237	0.078
Maximal stent diameter, mm	−0.786	−1.863–0.29	0.15
Treatment with ezetimibe, no vs. yes	−4.095	−9.173–0.981	0.11
Insulinotherapy, no vs. yes	−4.556	−9.15–0.038	0.05
Creatinine, µmol/L	−0.114	−0.249–0.02	0.09
Platelets count, $*10^3$/µL	−0.036	−0.075–0.002	0.06
Red cells count, $*10^6$/µL	5.14	−0.823–11.103	0.09
Total cholesterol, mmol/L	1.806	−0.956–4.569	0.19
LDL chol, mmol/L	2.184	−0.978–5.346	0.17
TGL, mmol/L	5.364	−0.506–11.235	0.07
NT-pro BNP, pg/mL	−0.001	−0.002–0.0001	0.07
Summary stent’s length, mm	0.112	−0.013–0.237	0.07
Maximal stent diameter, mm	−0.786	−1.863–0.29	0.15

CABG, coronary artery bypass grafting; COPD, chronic obstructive pulmonary disease; CVD, cardiovascular disease; GKS, glicocorticosteroids; LDL, low-density lipoproteins; NT-proBNP, N-terminal-pro-B-type natriuretic peptide; TGL, triglycerides; STS, Society of Thoracic Surgeons.
